# Induction of myelopoiesis by *Candida dubliniensis* drives protective trained immunity against sepsis in a Card9-dependent manner

**DOI:** 10.1128/mbio.02906-25

**Published:** 2025-10-31

**Authors:** Shannon Esher Righi, Elizabeth A. Lilly, Amanda J. Harriett, Patrick W. Daly, MaryJane Jones, Chad Steele, Mairi C. Noverr, Paul L. Fidel

**Affiliations:** 1 Department of Microbiology & Immunology, Tulane University School of Medicine 12255, New Orleans, Louisiana, USA; 2 Center of Excellence in Oral and Craniofacial Biology, Louisiana State University Health School of Dentistry 116781 , New Orleans, Louisiana, USA; Instituto Carlos Chagas, Curitiba, Brazil

**Keywords:** trained immunity, *Candida*, myelopoiesis, MDSC, sepsis

## Abstract

Trained immunity is a long-term functional reprogramming of innate cells that allows for the formation of immune memory akin to adaptive responses, but with a more rapid nonspecific response. We recently identified an anti-inflammatory trained immunity response, termed trained tolerogenic immunity (TTI), induced by low virulence *Candida dubliniensis,* that protects against acute lethal polymicrobial sepsis via myeloid-derived suppressor cells (MDSCs). Trained immunity and MDSCs arise following hematopoietic stem and progenitor cell (HSPC) expansion in the bone marrow. Here, we show that *C. dubliniensis* induces inoculum-dependent protection and HSPC expansion, whereas depletion of HSPCs following *C. dubliniensis* immunization abrogates protection. We further show that myeloid progenitors and putative MDSCs are increased in the bone marrow following *C. dubliniensis* immunization, and that HSPC depletion results in reduced MDSC influx into the peritoneal cavity following lethal challenge. Bone marrow cytokine analysis revealed that mediators associated with MDSC development and trafficking, including G-CSF, CXCL2, and CCL2, are upregulated following *C. dubliniensis* immunization. Finally, we find that the C-type lectin receptor adaptor protein Card9 is required for *C. dubliniensis*-induced protection and HSPC expansion, but not fungal trafficking to the bone marrow. Taken together, these results suggest that Card9-dependent fungal recognition in the bone marrow drives myelopoiesis and the induction of immunosuppressive MDSCs that are protective against lethal inflammation and sepsis.

**IMPORTANCE:**

Cells of the innate immune system can be “trained” by inducers to have enhanced memory responses, a phenomenon known as trained immunity. We recently identified an anti-inflammatory training response that is induced by low virulence fungal species (i.e., *Candida dubliniensis*) and is protective against acute lethal polymicrobial sepsis. Trained immunity inducers, including *C. dubliniensis,* can access the bone marrow and direct hematopoietic responses. Here, we demonstrate that protection is correlated with *C. dubliniensis*-induced bone marrow expansion, which directs a myeloid bias in the bone marrow and ultimately results in the expansion of protective myeloid-derived suppressor cells. Involvement of the C-type lectin receptor adaptor protein Card9 in the protective response suggests fungal recognition in the bone marrow drives this response. These findings offer new insights into how trained immunity inducers direct differential outcomes, which will inform the development of novel immunotherapeutics to exploit the full spectrum of trained immune responses.

## INTRODUCTION

Immunological memory is no longer restricted to cells of the adaptive immune system, but now also firmly established among innate cells. Accordingly, accumulating evidence shows that myeloid cells can be “trained” by inducers to have an enhanced response to secondary challenge, a phenomenon known as trained immunity (TI) (1–3). We previously showed that immunization with *Candida dubliniensis*, as well as other low virulence *Candida* species and abiotic fungal cell wall components, can induce innate immune protection against lethal challenge in various murine models of acute fungal/bacterial sepsis (4, 5). In contrast to prototypical TI inducers, such as the Bacillus Calmette-Guérin (BCG) vaccine and fungal β-glucan, which are associated with trained monocytes/macrophages and enhanced proinflammatory functions, including heightened pathogen killing, *C. dubliniensis*-induced protection involves anti-inflammatory Gr-1^+^ myeloid-derived suppressor cells (MDSCs) that mediate protection against sepsis via the suppression of lethal inflammation (i.e., cytokine storm) (6). We have shown that this protection is long-lived (up to 60 days post-immunization), maintained across multiple lethal challenges, and cross-protective between different immunization and challenge routes of administration (intraperitoneal and intravenous) (4, 7, 8). We have posited that this is a complementary form of trained immunity, which we have termed trained tolerogenic immunity (TTI) (9). Unlike the responses induced by BCG and β-glucan that serve to limit microbial growth and resolve infectious insults, the outcomes of *C. dubliniensis*-mediated trained tolerogenic immune responses serve to limit host-mediated damage and resolve inflammatory insults. Thus, TTI may represent a novel therapeutic strategy to broadly target inflammatory responses in a pathogen-agnostic manner.

While it was previously unclear how relatively short-lived mature innate immune cells maintain their memory over long periods of time, recent work has highlighted the role of hematopoietic stem and progenitor cells (HSPCs) in the bone marrow in conferring memory responses. Referred to as central trained immunity (10), HSPCs can be reprogrammed to produce more myeloid progenitors, thereby driving a myelopoietic bias and the production of trained progeny. HSPCs can respond to signals derived from the bone marrow microenvironment itself, distal inflammatory sites, and directly to infectious agents (11). To that end, HSPCs, particularly early progenitor cells, express functional pattern recognition receptors (PRRs) (12), and TI inducers, such as BCG, have been shown to directly access the bone marrow compartment (13). We have similarly shown that *C. dubliniensis* can access the bone marrow, and that the level of bone marrow infiltration by different *Candida* species correlates with their ability to induce TTI against sepsis (7). Furthermore, *in vitro* studies have demonstrated that stimulation of HSPCs with Dectin-1 ligands, including *Candida albicans* or β-glucan, results in differentiated macrophages and neutrophils that produce high levels of proinflammatory cytokines, while HSPCs stimulated with toll-like receptor (TLR) ligands resulted in cells with reduced cytokine production (14–16), demonstrating that interaction with different HSPC PRRs can drive distinct training outcomes. This suggests that the initial training stimuli can direct the type and nature of the myeloid cell that arises.

MDSCs are a heterogeneous population of pathologically activated immature myeloid cells that arise from expanded myeloid progenitor populations in the bone marrow (17), similar to the induction of TI. We previously documented the presence of mature, functionally immunosuppressive MDSCs in the peritoneal cavity of *C. dubliniensis* immunized mice following septic challenge (6), yet, how these cells are induced and develop following immunization remains unclear. Myeloid cell expansion in the bone marrow during MDSC development is driven by growth factors and inflammatory mediators, generally in response to chronic conditions and persistent inflammation (18). Given that *C. dubliniensis* can access the bone marrow compartment similar to other TI inducers, and that MDSCs arise from an expanded myeloid progenitor population in the bone marrow similar to the induction of TI, we hypothesized that *C. dubliniensis* may be interacting with HSPCs in the bone marrow to preferentially produce trained MDSCs.

The present study aimed to investigate HSPC dynamics, MDSC development, and PRR recognition associated with *C. dubliniensis* immunization and migration to the bone marrow. We show that *C. dubliniensis* immunization results in an expansion of the overall HSPC population, as well as the myeloid progenitor population and putative MDSCs. Importantly, bone marrow expansion, as well as *C. dubliniensis*-mediated protection against sepsis, requires the C-type lectin receptor adaptor protein Card9, suggesting that fungal recognition is an important component of this process. Together, these results support a model by which *C. dubliniensis* directly interacts with cells within the bone marrow to induce training, modulate myelopoiesis, and drive immunosuppressive MDSC production that is protective against lethal sepsis.

## RESULTS

### *C. dubliniensis* protection is associated with HSPC expansion

**Fig 1:**
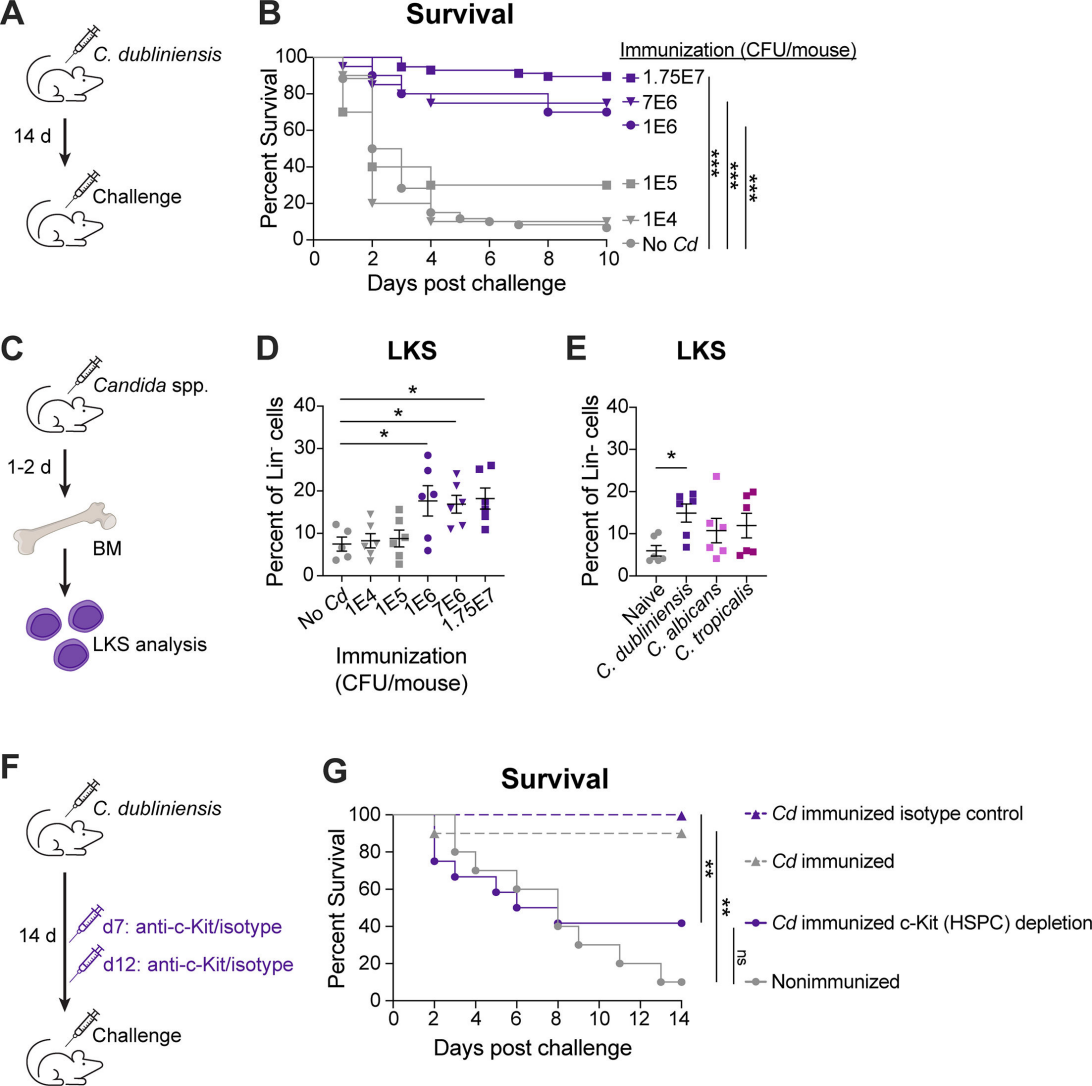
*C. dubliniensis* induces bone marrow HSPC expansion that is required for protection. (**A**) Experimental design: WT Swiss Webster mice were immunized IP with increasing *C. dubliniensis* inocula (1 × 10^4^–1.75 × 10^7^ cells), followed by lethal IP polymicrobial sepsis challenge (2.1 × 10^7^ *C. albicans* cells + 8 × 10^7^ *S. aureus* cells) 14 days later. (**B**) Survival following lethal sepsis challenge. *n* = 10/group; representative of two independent experiments. ***P < 0.001, log-rank test with Holm-Sidak multiple comparisons test. (**C**) Experimental design: bone marrow (BM) cells were collected from mice 1–2 days post-inoculation with *Candida* spp., and total HSPCs (LKS cells, Lin^−^ c-Kit^+^, Sca-1^+^) were assessed by flow cytometry. (**D**) LKS cell expansion following immunization with increasing *C. dubliniensis* inocula. *n* = 5–6/group, combined from two experiments. (**E**) LKS cell expansion following inoculation with 1.75 × 10^7^ *C. dubliniensis*, *C. albicans*, or *Candida tropicalis* cells. *n* = 6/group, combined from two experiments. *P < 0.05, one-way analysis of variance (ANOVA) with Dunnett’s multiple comparisons test. Data are mean ± s.e.m. Representative full gating strategy illustrated in Fig. S2A and B. (**F**) Experimental design: WT Swiss Webster mice were immunized IP with 1.75 × 10^7^ *C. dubliniensis* cells, followed by IV administration of 500 µg of anti-c-Kit (clone 2B8) or isotype control antibody on day 7 and day 12, and lethal IP polymicrobial sepsis challenge on day 14. (**G**) Survival following c-Kit (HSPC) depletion and lethal sepsis challenge. *n* = 10–14/group. **P < 0.01, log-rank test with Holm-Sidak multiple comparisons test.

We previously documented that *Candida* species can access the bone marrow compartment within 24 h of intraperitoneal (IP) inoculation, and further showed that protection was correlated with the level of bone marrow access by different *Candida* species; mice immunized with *C. dubliniensis* had the highest bone marrow burdens and exhibited the highest levels of survival (7). To identify the minimum immunization dose required for *C. dubliniensis*-induced protection, we inoculated mice with decreasing amounts of live fungus and tested survival after challenge in our polymicrobial sepsis model (Fig. 1A; lethal *C. albicans + Staphylococcus aureus* intra-abdominal coinfection; see Materials and Methods and references 19, 20). Protection was maintained when the standard immunization inocula (1.75 × 10^7^ cells/mouse) was reduced by up to 17.5-fold (1 × 10^6^ cells/mouse) but was lost when the inocula was reduced below 1 × 10^5^ cells/mouse (Fig. 1B).

Based on our previous data demonstrating a correlation between *C. dubliniensis* bone marrow access and TTI protection (7), and studies establishing the bone marrow as the site of central trained immunity (21), we hypothesized that *C. dubliniensis* immunization/bone marrow access influences HSPC populations. To test this, we measured the levels of total, uncommitted HSPCs (lineage^−^ c-Kit^+^ Sca-1^+^, referred to as LKS cells) in the bone marrow 1–2 days following *C. dubliniensis* immunization (Fig. 1C). LKS cells were increased following *C. dubliniensis* immunization, but only at those inocula that resulted in protection (Fig. 1D). *C. albicans* and *C. tropicalis*, which provide intermediate and low levels of protection in our model (4), respectively, did not induce a significant expansion of LKS cells above naïve mice at the same time point (1–2 days) when given at the standard inocula (1.75 × 10^7^ cells/mouse) (Fig. 1E). Collectively, these data show a *C. dubliniensis* dose-dependent effect on protection and HSPC expansion in the bone marrow.

To directly test the role of HSPC expansion on the induction of protective trained tolerogenic immunity, we used an antibody against c-Kit to deplete the HSPC population following *C. dubliniensis* immunization (Fig. 1F). Following IV antibody administration at 7 and 12 days post-immunization (dpi) and lethal polymicrobial sepsis challenge at 14 dpi, mice that received anti-c-Kit antibody displayed significantly higher mortality compared to those that received the isotype control antibody (42% vs 100%, respectively; *P* = 0.0032; Fig. 1G). Follow-up studies with earlier depletion time points and a second anti-c-Kit antibody clone (ACK2 vs 2B8) revealed a similar increase in mortality among mice receiving the c-Kit depletion antibody, although non-significant (Fig. S1). Together, these results indicate a significant role for the expanded HSPC population in *C. dubliniensis*-mediated protection.

### *C. dubliniensis* promotes myelopoiesis in the bone marrow

**Fig 2:**
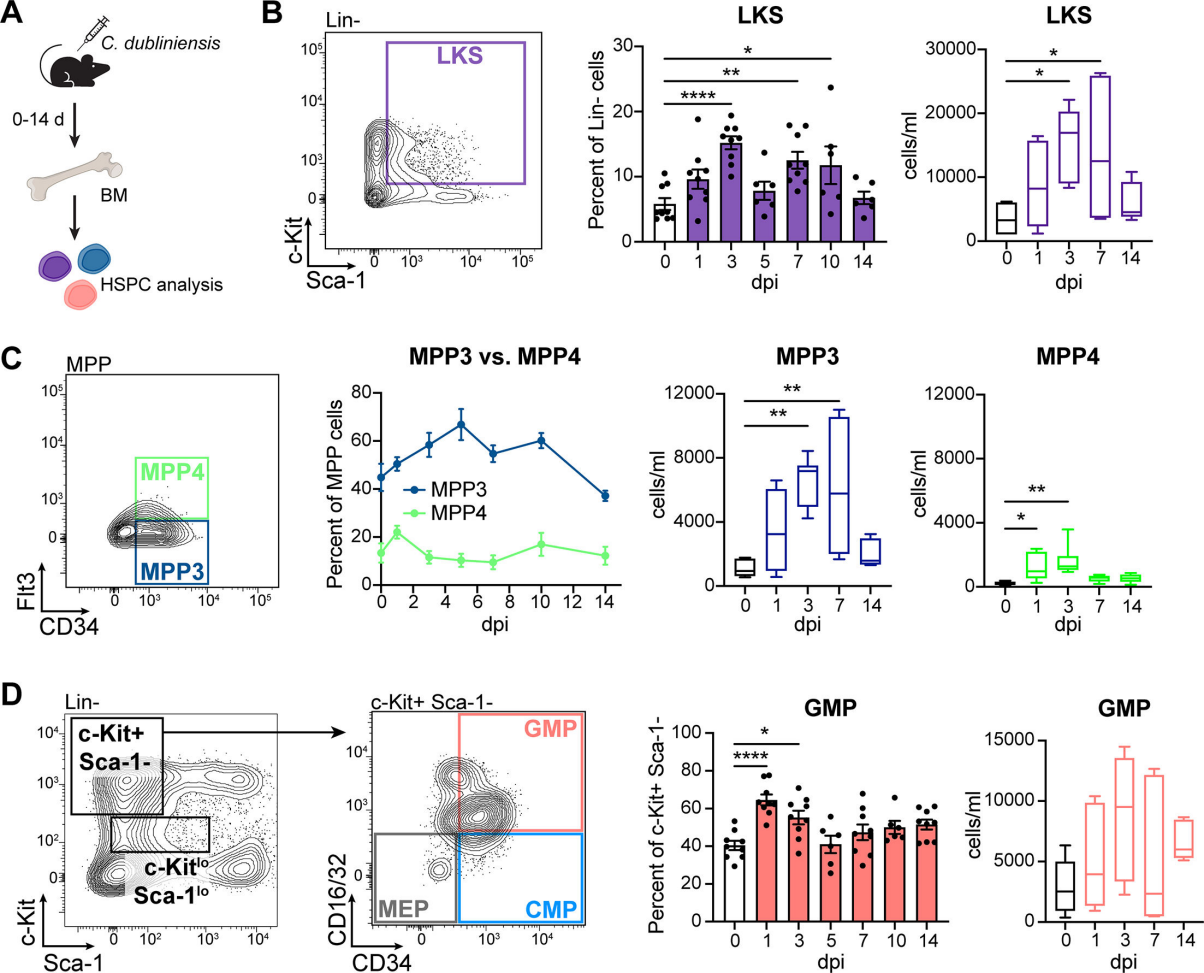
The bone marrow is biased toward myelopoiesis following *C. dubliniensis* immunization. (**A**) Experimental design: BM cells were isolated from WT C57BL/6 at the indicated time points post-IP immunization with 1.75 × 10^7^ *C. dubliniensis* cells and HSPCs were assessed by flow cytometry. (**B**) Total HSPCs (LKS cells; Lin^−^ c-Kit^+^ Sca-1^+^). (**C**) Myeloid-biased multipotent progenitor 3 (MPP3; LKS CD150^−^ CD48^+^ CD34^+^ Flt3^−^) vs lymphoid-biased MPP4 (LKS CD150^−^ CD48^+^ CD34^+^ Flt3^+^) cells. (**D**) Granulocyte-macrophage progenitor (GMP; Lin^−^ c-Kit^+^ Sca-1^−^ CD34^+^ CD16/32^−^) cells. Representative gating (left), cell percentages of parent population (middle; *n* = 6–9/group, combined from two to three experiments), and cell numbers expressed as cells/mL (right; *n* = 6/group, combined from two experiments). * *P* < 0.05, **P < 0.01, ****P < 0.0001, one-way ANOVA with Dunnett’s multiple comparisons test. Percentage data are mean ± s.e.m.; cell number data are boxplots showing 25–75 percentiles, medians, and whiskers spanning min–max. Representative full gating strategies and additional HSPC populations illustrated in Fig. S2 and S3.

Based on similarities to HSPC expansion seen with prototypical TI inducers (13, 22), we hypothesized that *C. dubliniensis* immunization induces protection by reprogramming HSPCs in the bone marrow to support myelopoiesis and the production of trained myeloid cells. To further characterize the bone marrow compartment following *C. dubliniensis* immunization, we collected bone marrow cells from C57BL/6 mice over time following immunization with the standard inocula and assessed HSPC subpopulations in detail by flow cytometry (Fig. 2; Fig. S2). Similar to above, LKS cells were increased in this mouse background by nearly twofold at 1 dpi compared to naïve mice (0 dpi), peaking in both proportion and cell number at 3 dpi, and remaining just above baseline at 14 dpi (Fig. 2B). Assessment of LKS HSPC subsets revealed no changes in the proportions of self-renewing long-term hematopoietic stem cells (LT-HSC; LKS CD150^+^ CD48^−^), pluripotent short-term hematopoietic stem cells (ST-HSC; LKS CD150^−^ CD48^+^), or multipotent progenitor cells (MPP; LKS CD150^−^ CD48^+^) following immunization; however, these populations were increased in absolute number compared to naïve mice, particularly at 3 dpi (Fig. S3A through C). There was also a significant increase in the absolute numbers of both the myeloid-biased MPP3 (LKS CD150^−^ CD48^+^ CD34^+^ Flt3^−^) and lymphoid-biased MPP4 (LKS CD150^−^ CD48^+^ CD34^+^ Flt3^+^) subsets following immunization, with only the myeloid-biased MPP3 subset increasing in frequency (Fig. 2; Fig. S3D and E), indicating a skewing toward the myeloid lineage.

Oligopotent common myeloid progenitor (CMP; Lin^−^ c-Kit^+^ Sca-1^−^ CD34^+^ CD16/32^−^) cell proportions and numbers were unchanged following immunization (Fig. S3F and G). By contrast, downstream granulocyte macrophage progenitors (GMP; Lin^−^ c-Kit^+^ Sca-1^−^ CD34^+^ CD16/32^+^) were increased (Fig. 2D), while megakaryocyte/erythroid progenitors (MEP; Lin^−^ c-Kit^+^ Sca-1^−^ CD34^−^ CD16/32^−^) were decreased concurrently (Fig. S3F and H), indicating an expansion of CMP-derived GMPs at the expense of MEPs. Common lymphoid progenitor (CLP; Lin^−^ c-Kit^lo^ Sca-1^lo^ IL-7Rα^+^) cell proportions and absolute numbers were also unchanged overall throughout the time course (Fig. S3I). Together with the MPP3 myeloid lineage skewing above, these data demonstrate that *C. dubliniensis* IP immunization enhances myelopoiesis in the bone marrow.

### Putative MDSCs are expanded in the bone marrow following immunization

**Fig 3:**
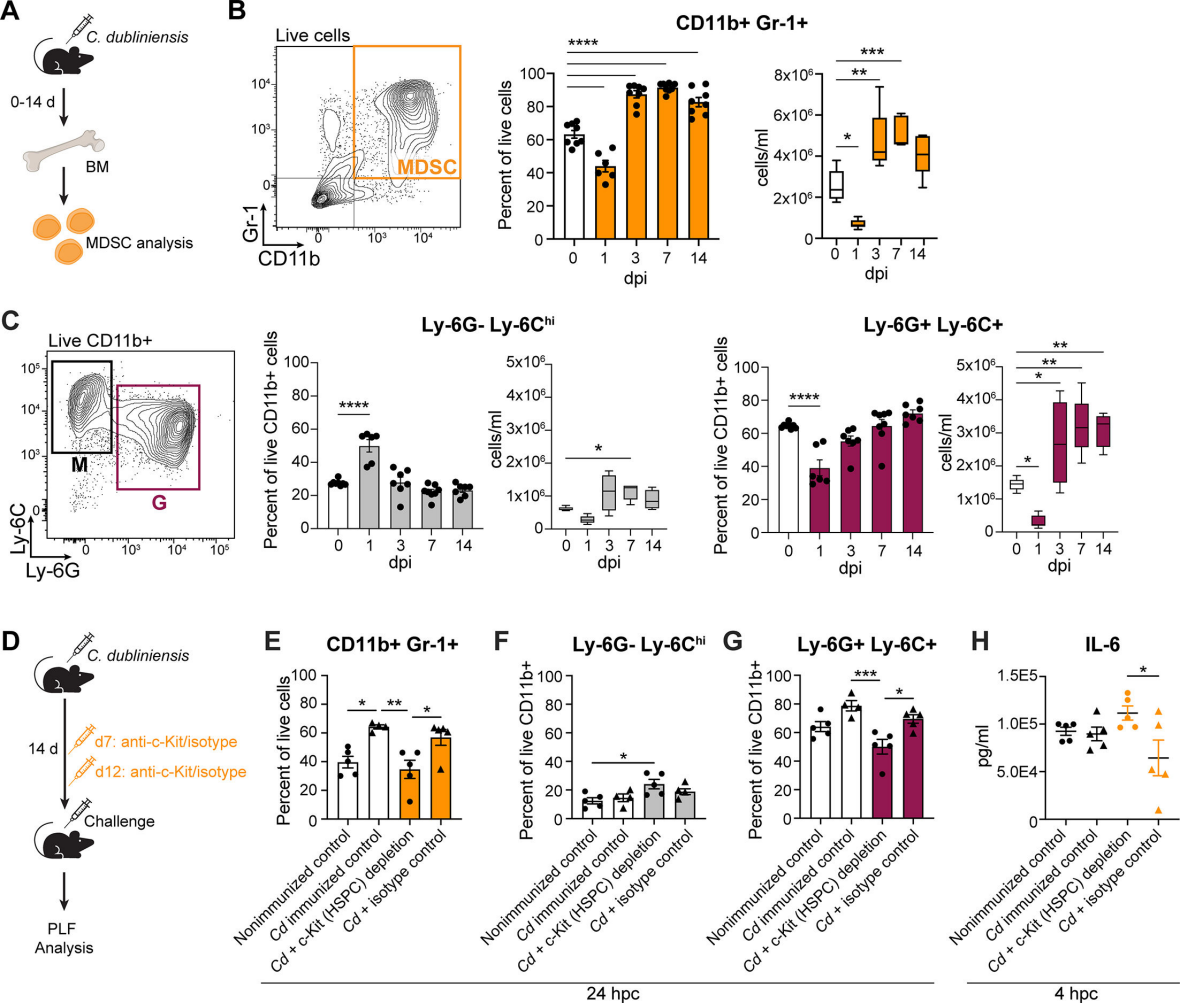
Expansion of putative MDSCs is induced by *C. dubliniensis* immunization and dependent on HSPCs. (**A**) Experimental design: BM cells were isolated from WT C57BL/6 mice at the indicated time points post-IP immunization with 1.75 × 10^7^ *C. dubliniensis* cells and putative MDSCs were assessed by flow cytometry. (**B**) CD11b^+^ Gr-1^+^ cells (putative MDSCs). (**C**) CD11b^+^ Ly-6G^−^ Ly-6C^hi^ cells (putative monocytic MDSC subset) vs CD11b^+^ Ly-6G^+^ Ly-6C^+^ cells (putative granulocytic MDSC subset). Representative gating (left), cell percentages of parent population (middle; *n* = 6–8/group, combined from two experiments), and cell numbers expressed as cells/mL (right; *n* = 5/group). *P < 0.05, **P < 0.01, ***P < 0.001, ****P < 0.0001, one-way ANOVA with Dunnett’s multiple comparisons test. Percentage data are mean ± s.e.m.; cell number data are boxplots showing 25–75 percentiles, medians, and whiskers spanning min–max. Representative full gating strategies illustrated in Fig. S4. (**D**) Experimental design: WT C57BL/6 mice were immunized as above, followed by IP administration of 500 µg anti-c-Kit or isotype control antibody on day 7 and day 12, and lethal IP polymicrobial sepsis challenge on day 14. Peritoneal lavage fluid (PLF) was collected at 4 and 24 h post-challenge (hpc) for analysis. (**E**) CD11b^+^ Gr-1^+^ cells (putative MDSCs), (**F**) CD11b^+^ Ly-6G^−^ Ly-6C^hi^ cells (putative M-MDSC subset), and (**G**) CD11b^+^ Ly-6G^+^ Ly-6C^+^ cells (putative G-MDSC subset) in the PLF 24 hpc, determined by flow cytometry analysis. (**H**) IL-6 levels in the PLF 4 hpc, determined by enzyme-linked immunosorbent assay (ELISA). *n* = 5/group. *P < 0.05, **P < 0.01, ***P < 0.001, one-way ANOVA with Tukey’s multiple comparisons test. Data are mean ± s.e.m.

Our previous work established the presence of increased levels of immunosuppressive MDSCs in the peritoneal cavity of immunized mice during lethal sepsis challenge (6). MDSCs arise from expanded immature myeloid precursor populations, including GMPs, in the bone marrow, before trafficking to peripheral sites (18). In addition to functional MDSCs in the peritoneal cavity, we also documented increased putative MDSCs (CD11b^+^ Gr-1^+^), particularly the granulocytic G-MDSC subset (CD11b^+^ Ly-6G^+^ Ly-6C^+/lo^), in the bone marrow of immunized mice immediately prior to challenge (6). To assess the kinetics of putative MDSC expansion in the bone marrow following immunization, we measured CD11b^+^ Gr-1^+^ cells over time, observing an initial decrease at 1 dpi, followed by an expansion of these cells both in proportion and number (Fig. 3A and B). Levels of putative G-MDSCs followed a similar pattern, with significantly increased cell numbers by 14 dpi, while the monocytic M-MDSC (CD11b^+^ Ly-6G^−^ Ly-6C^hi^) subset returned to baseline levels by 14 dpi (Fig. 3C).

To directly assess the role of bone marrow expansion on the downstream development of functional MDSCs, we measured the accumulation of CD11b^+^ Gr-1^+^ cells in the peritoneal cavity of HSPC-depleted mice following sepsis challenge (Fig. 3D). Here, we observed an increase in both total putative MDSCs and the granulocytic G-MDSC subset in the peritoneal cavity of *C. dubliniensis* immunized mice 24 h after lethal sepsis challenge. By contrast, in the peritoneal cavity of immunized, anti-c-Kit antibody-treated mice, the levels of total putative MDSCs and G-MDSCs were similar to that observed in nonimmunized control mice (Fig. 3E through G). Furthermore, when we assayed the representative proinflammatory cytokine, IL-6, in the peritoneal cavity 4 h after lethal challenge, we observed increased levels in immunized, anti-c-Kit antibody treated mice compared to isotype control antibody treated mice (Fig. 3H).

### Cytokines associated with MDSC development and trafficking are increased in the bone marrow following *C. dubliniensis* immunization

**Fig 4:**
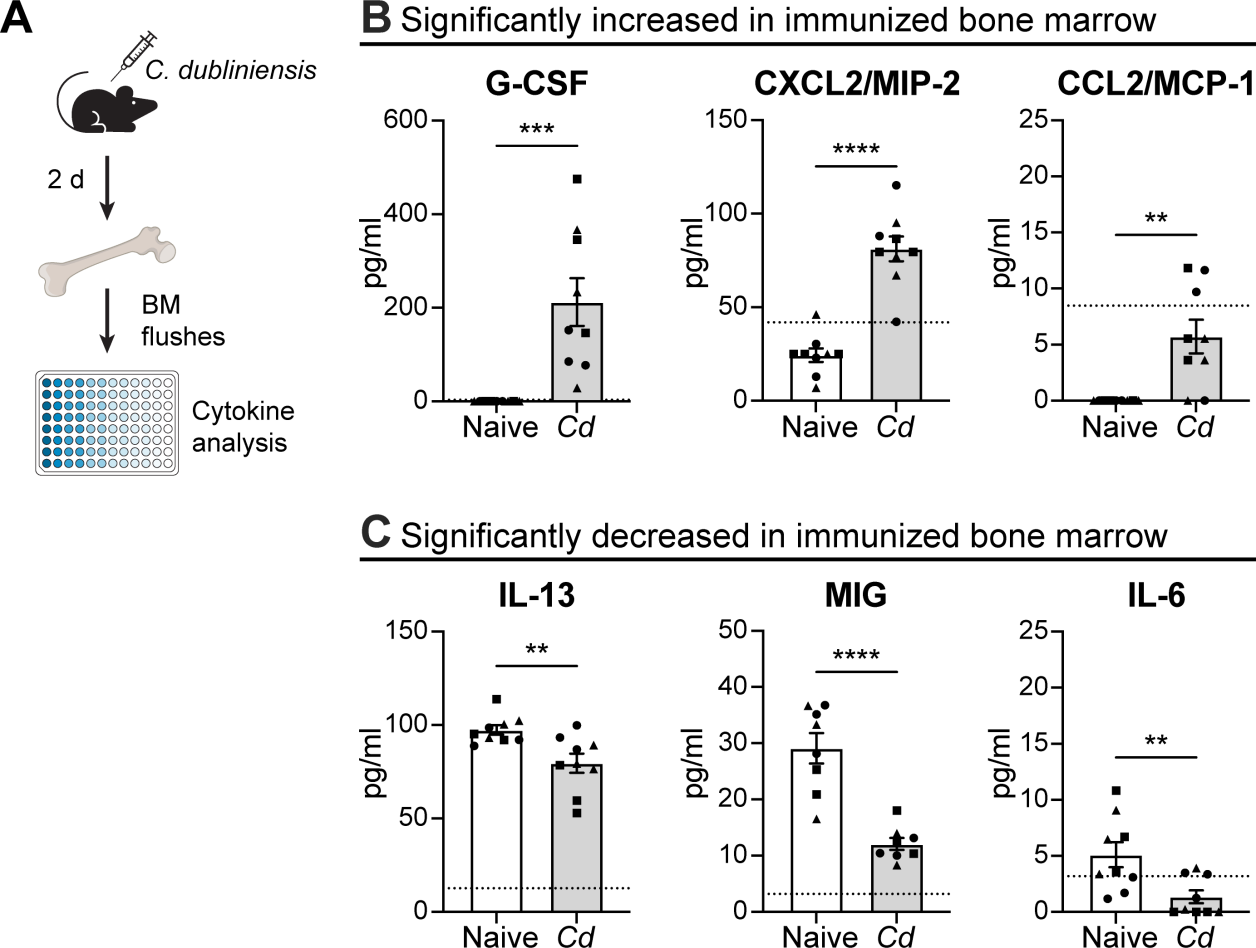
Cytokine modulations in the bone marrow of *C. dubliniensis* immunized mice. (**A**) Experimental design: femurs from WT C57BL/6 mice were isolated 2 days post-IP immunization with 1.75 × 10^7^ *C. dubliniensis* cells and flushed to obtain bone marrow supernatants that were analyzed by multiplex ELISA. (**B**) Significantly increased (G-CSF, CXCL2/MIP-2, CCL2/MCP-1) and (**C**) significantly decreased (IL-13, CXCL9/MIG, IL-6) cytokine levels in naïve and *C. dubliniensis* immunized mouse bone marrow. *n* = 9/group, combined from three experiments (symbols represent independent experiments). **P < 0.01, ***P < 0.001, ****P < 0.0001, two-tailed *t*-test. Data are mean ± s.e.m. Dashed line indicates lower limit of detection.

To understand the early responses induced in the bone marrow by *C. dubliniensis* immunization, we collected bone marrow extracellular fluid 2 days post-immunization for analysis by multiplex enzyme-linked immunosorbent assay (ELISA) (Fig. 4A). Among those tested, G-CSF, CXCL2/MIP-2, and CCL2/MCP-1 were significantly increased in bone marrow extracellular fluid from immunized mice compared to naïve mice (Fig. 4B). All three of these factors have been implicated in aspects of MDSC development and trafficking/recruitment, with G-CSF considered one of the main growth factors promoting myelopoiesis and MDSC expansion (23), and both CXCL2/MIP-2 and CCL2/MCP-1 reportedly implicated in MDSC mobilization, recruitment, and function in various types of cancer (24). IL-1β, IL-12p70, and CXCL1/KC were also significantly increased in immunized bone marrow; however, their levels were below the limit of detection (Fig. S5A). Interestingly, most cytokines tested were at or below the limit of detection, with the exception of IL-9, IL-1α, and CCL11/Eotaxin, which were uniformly elevated in both naïve and immunized samples (Fig. S5). IL-13, CXCL9/MIG, and IL-6 were the only cytokines significantly decreased in the bone marrow of immunized mice (Fig. 4C).

### Trained immunity induction by *C. dubliniensis* requires Card9

**Fig 5:**
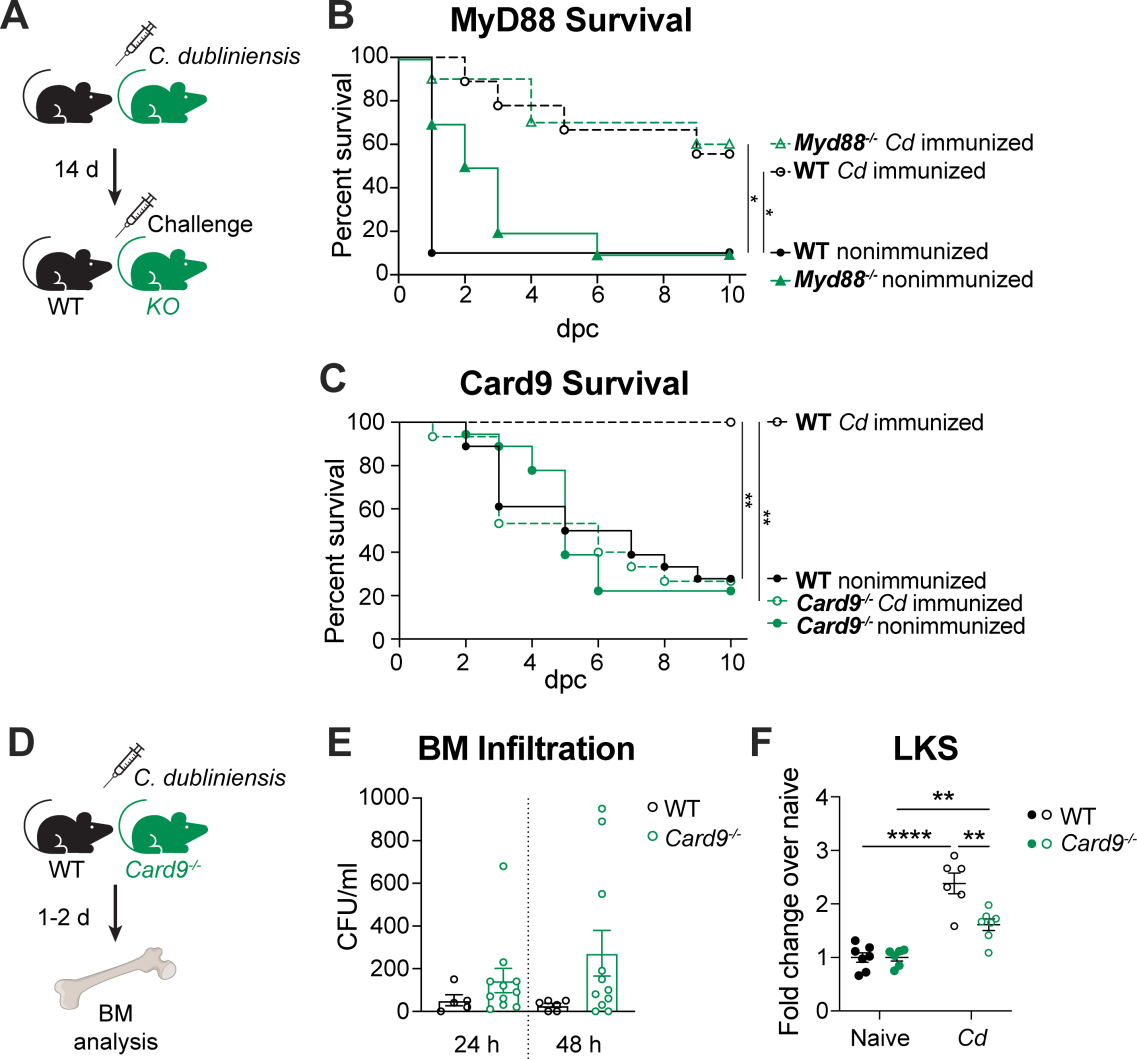
Card9 is required for protection and bone marrow expansion. (**A**) Experimental design: WT and *Myd88^−/−^* or *Card9^−/−^* C57BL/6 mice were immunized IP with *C. dubliniensis* (1.75 × 10^7^ cells in B or 7 × 10^6^ cells in C), followed by lethal IP polymicrobial sepsis challenge (1.75 × 10^7^ *C. albicans* in B or 7 × 10^6^ *C. albicans* in C + 8 × 10^7^ *S. aureus* cells) 14 days later. (**B**) Survival of *Myd88^−/−^* mice following lethal sepsis challenge. *n* = 9–10/group. (**C**) Survival of *Card9^−/−^* mice following lethal sepsis challenge. *n* = 15–18/group, combined from two experiments. *P < 0.05, **P < 0.01, log-rank test with Holm-Sidak multiple comparisons test. (**D**) Experimental design: bone marrow from WT and *Card9^−/−^* C57BL/6 mice was isolated 1–2 days post-IP immunization with 1.75 × 10^7^ *C. dubliniensis* cells. (**E**) *C. dubliniensis* BM infiltration. *n* = 5–6/group (WT) and *n* = 11/group (*Card9*^−/−^, combined from two experiments). *ns*, WT vs *Card9^−/−^* at either time point, one-way ANOVA with Sidak’s multiple comparisons test. Data are mean ± s.e.m. (**F**) LKS cell expansion. *N* = 6–7/group, combined from two experiments. **P < 0.01, ****P < 0.0001, two-way ANOVA with Sidak’s multiple comparison test. Data are expressed as fold change over naïve mice.

HSPCs express functional PRRs that can recognize and respond to microorganisms and TI inducers (12). Furthermore, MDSC expansion during polymicrobial sepsis was found to be dependent on the TLR adaptor protein MyD88 (25). Therefore, we hypothesized that HSPC expansion and ultimately protection may be driven by PRR recognition of *C. dubliniensis* in the bone marrow. To test this, we immunized mice lacking MyD88 and tested survival in our polymicrobial sepsis model (Fig. 5A). Immunized *Myd88*-deficient mice maintained a level of protection similar to WT mice (Fig. 5B), indicating that *C. dubliniensis* is likely not recognized by a TLR. Considering the role that C-type lectin receptors (CLRs) play in fungal recognition (26), we next tested the CLR adaptor protein Card9. In contrast to *Myd88*-deficient mice, immunized *Card9*-deficient mice completely lost protection and resembled nonimmunized mice (Fig. 5C), indicating that CLR recognition is required for *C. dubliniensis*-induced TTI. To differentiate a role for Card9 in *C. dubliniensis* trafficking to the bone marrow from a role in *C. dubliniensis*-induced HSPC expansion, we assessed bone marrow phenotypes shortly after immunization (Fig. 5D). When we plated bone marrow from immunized mice for CFUs, we observed a similar level of *C. dubliniensis* between WT and *Card9*-deficient mice (Fig. 5E), demonstrating that lack of access to the bone marrow by *C. dubliniensis* was not a factor in the loss of protection in these mice. Conversely, to test whether Card9 is required for bone marrow expansion, we measured LKS cells following *C. dubliniensis* immunization. To account for any differences in baseline LKS levels, we measured fold change over naïve mice among WT and *Card9*-deficient mice. While there was a significant increase in LKS cells for all immunized mice, this increase was significantly less in *Card9* deficient mice (Fig. 5F), suggesting that CLR signaling is required for recognizing and inducing a full response to *C. dubliniensis* in the bone marrow.

## DISCUSSION

The concept of central TI, or innate immune training among myeloid precursor cells in the bone marrow, has recently gained considerable attention as a mechanism by which immune memory can persist in mature, relatively short-lived innate cells. TI inducers, such as BCG, gain access to the bone marrow compartment to induce changes in the HSPC populations, including expanding myeloid-biased progenitor populations in favor of myelopoiesis (27). Similarly, MDSCs arise initially from an expanded myeloid progenitor population in the bone marrow (28). We and others have demonstrated that *Candida* species, including *C. dubliniensis*, can induce MDSC accumulation *in vitro* and *in vivo* (6, 29, 30); however, whether HSPC changes associated with central trained immunity can drive MDSC differentiation had not yet been investigated. We previously reported that *C. dubliniensis* traffics to the bone marrow compartment following IP immunization (7), leading to the hypothesis that *C. dubliniensis* may be influencing HSPCs similar to other TI inducers. We therefore sought to define HSPC dynamics *in vivo* following *C. dubliniensis* immunization. Through detailed bone marrow phenotyping studies, we demonstrate a novel connection between *C. dubliniensis*-induced expansion of the HSPC compartment, MDSC development, and TTI protection against lethal sepsis of intra-abdominal origin.

Like other TI inducers, we show that *C. dubliniensis* drives an inoculum-dependent expansion of HSPCs. Specifically, we observed an increase in uncommitted LKS cells, myeloid-biased MPP3 cells, and downstream GMPs following *C. dubliniensis* immunization. We did not observe the same level of LKS cell expansion when mice were inoculated with *C. albicans* or *C. tropicalis*, which aligns with our previous data demonstrating that these high virulence *Candida* spp. cannot provide the same level of protection as *C. dubliniensis*, even at sublethal inocula, and supports that protection is correlated with bone marrow expansion (4, 7, 31). *C. albicans* has been previously reported to induce HSPC proliferation and an expansion of Lin- and LKS cells *in vivo* (32); however, these prior studies found expansion only when mice were given a low-level infection with inocula over 40× lower than that employed in our studies. Conversely, we reported that the *C. albicans* inocula used in our studies induces considerable damage in the bone marrow, and that protection is negatively correlated with such damage (31), which could also explain the discrepancy in the LKS expansion data. Nevertheless, the lack of HSPC expansion with virulent *Candida* spp. also suggests that this is a specific response to *C. dubliniensis*, rather than a generalized emergency myelopoiesis response, although the distinction between emergency myelopoiesis and early events in trained immunity is still not well understood (33).

Using antibodies against c-Kit, we depleted the expanded HSPC population following *C. dubliniensis* immunization and demonstrated a significant abrogation of protection in the absence of this population. Initial studies were performed using the 2B8 c-Kit antibody clone, which reportedly only results in a partial inhibition of c-Kit signaling. Therefore, we performed follow-up studies using the more commonly accepted ACK2 clone and carried out the depletions over a shorter time period between immunization and challenge to ensure that recently trained cells were being depleted and not given time to replenish. While not statistically significant, we still observed a similar reduction in protection among HSPC-depleted mice, indicating at least a partial role for this expanded population. While the ACK2 antibody clone efficiently depletes HSCs, its impact on other c-Kit expressing progenitor cells, particularly GMPs and especially at early time points, is less pronounced (34). Additional studies will be required to delineate the specific progenitor cell populations that respond to *C. dubliniensis*. Accordingly, future studies will aim to deplete individual cell populations at various time points before and after *C. dubliniensis* immunization.

Another intriguing possibility suggested by the partial effect of HSPC depletion is the potential involvement of mesenchymal stromal cells (MSCs). These cells have been shown to undergo trained immunity themselves (35), in addition to playing both positive and negative roles in training HSPCs (36, 37). Furthermore, MSCs secrete soluble factors that can act in a paracrine manner on HSPCs to drive myeloid cell differentiation and granulopoiesis (38–40), as well as MDSC differentiation (41). Of particular interest to our study, MSCs secrete CCL2, which can induce the emigration of monocytes out of the bone marrow (42), as well as reprogram mature macrophages to be more immunosuppressive and secrete IL-10 (43, 44), a key MDSC effector cytokine and one that we found to be required for *C. dubliniensis*-mediated protection (6). Importantly, PRR signaling has been implicated in MSC-induced HSPC changes (39, 40), suggesting that these cells might respond directly to *C. dubliniensis* in the bone marrow. Along those lines, MSCs have been reported to respond to a variety of fungi (reviewed in reference 45), including via PRR recognition and signaling (46). Based on these data, it is possible that MSCs recognize and respond to *C. dubliniensis* and secrete factors, such as CCL2, that act on HSPCs to induce myelopoiesis and the expansion of MDSCs. Critical next steps will include investigating changes in MSC phenotypes/populations and their requirements in the response to *C. dubliniensis*.

Regarding PRR recognition, we demonstrated a requirement for the C-type lectin receptor adaptor protein, Card9, in both LKS expansion and protection mediated by *C. dubliniensis*. This was independent of a role for Card9 in *C. dubliniensis* trafficking to the bone marrow, suggesting a direct interaction between a Card9-dependent receptor and *C. dubliniensis*. Supporting a direct interaction between *C. dubliniensis* and HSPCs, several studies have demonstrated that PRR signaling among HSPCs in response to *Candida* and other ligands can drive downstream differentiation of functionally distinct myeloid cells (14, 15, 32, 47–51). Most recently, Sobén et al. demonstrated that stimulation of HSPCs with Dectin-1 ligands or *C. albicans* results in trained neutrophils with enhanced proinflammatory and antimicrobial responses, while HSPCs stimulated with TLR ligands resulted in neutrophils with a blunted inflammatory response (16). BCG- and β-glucan-induced trained immunity have been associated with NOD-2 and Dectin-1, respectively (52–54), further pointing toward a PRR-TI inducer interaction. We found no role for MyD88, and while several intracellular TLRs signal independent of MyD88, our Card9 data strongly implicates a C-type lectin receptor in *C. dubliniensis* recognition in the bone marrow. In contrast to TI and *C. albicans* recognition, ongoing studies in our laboratory indicate that a non-Dectin-1 CLR is involved in *C. dubliniensis* recognition and the TTI response (P. W. Daly and S. E. Righi, unpublished data). Notably, the binding patterns of various CLR probes to *C. dubliniensis* cells were different from that of other *Candida* spp., including *C. albicans* (55). This suggests that CLR-specific recognition upstream of Card9 may drive the divergence between TI and TTI responses in our model. The specific CLR responsible for recognizing *C. dubliniensis* and directing HSPC expansion and TTI responses in the bone marrow, as well as the *C. dubliniensis* interacting ligand, is under active investigation by our laboratory.

Finally, we previously reported that immunosuppressive MDSCs, and specifically G-MDSCs, in the peritoneal cavity are required for *C. dubliniensis*-mediated protection against polymicrobial sepsis (6, 7). Here, we observed increased putative MDSCs/G-MDSCs in the bone marrow following *C. dubliniensis* immunization, combined with a reduction in the recruitment of these cells to the peritoneal cavity following lethal sepsis challenge in HSPC-depleted mice. Interestingly, the expansion of the putative CD11b^+^ Gr-1^+^ MDSC population in the bone marrow occurred following a rapid decrease in cells bearing these markers at 1 dpi. The concurrent expansion of myeloid progenitors observed in our HSPC analyses is suggestive of an initial purge of the typical granulocyte and monocyte populations, followed by their replacement/remodeling with MDSC precursors. Altogether, this suggests a model in which *C. dubliniensis* induces the expansion of myeloid progenitors and MDSC precursors in the bone marrow, which then traffic to the peritoneal cavity where they are activated and suppress sepsis-associated inflammation. In further support of this, we found significantly increased levels of cytokines/chemokines associated with hematopoiesis, granulopoiesis, and MDSC expansion/trafficking in the bone marrow following *C. dubliniensis* immunization. G-CSF is a central granulopoiesis and HSPC mobilization factor that can skew CMPs toward GMPs and is a major driver of neutrophil expansion (56, 57). It is also one of the key growth factors involved in MDSC expansion and differentiation (58, 59). Similarly, while CCL2 is most notable as a monocyte chemokine, it has also been implicated in driving the differentiation of GMPs into mature myeloid cells (38), and the expansion of MDSCs (60, 61). The chemokine CXCL2 is also an HSPC mobilization factor (62), and both the CCL2/CCR2 and CXCL2/CXCR2 signaling axes have been implicated in MDSC trafficking to tumors and sites of inflammation (61, 63–65). Specifically, CCL2/CCR2 drive MDSC mobilization from the bone marrow to the blood, while CXCL2/CXCR2 are involved in MDSC homing to tumors and other sites (reviewed in reference 66). Of particular note, CCL2 secretion specifically by MSCs has been implicated in MDSC homing from the bone marrow to tumor sites (67). Yet, key questions remain regarding the sources and targets of these cytokines/chemokines, as well as how their upregulation in the bone marrow translates to MDSC trafficking and activation in the peritoneal cavity. Studies are underway in our laboratory to investigate the roles of these cytokines/chemokines in *C. dubliniensis*-induced MDSC production and protection. Functional analyses of these putative bone marrow MDSCs are also ongoing, with a particular focus on the involvement of CLR/Card9 signaling. Future studies will aim to utilize *in vivo* fate mapping models to definitively track the origins and emergence of *C. dubliniensis*-induced MDSCs both in the bone marrow from progenitor cells and further as they traffic to the peritoneal cavity.

In conclusion, we have characterized changes in the bone marrow compartment that are induced by *C. dubliniensis*, dependent on Card9 signaling, and that drive myelopoiesis and the development of MDSCs that are protective against lethal polymicrobial sepsis of intra-abdominal origin. This induction of myelopoiesis mirrors that seen with traditional TI inducers; however, the resulting effector cells (MDSCs) and immune responses (anti-inflammatory) differ. These differential outcomes are key to what sets TTI responses apart from traditional TI responses and are critical to the protection against lethal inflammation. Collectively, these studies support a model in which *C. dubliniensis* directly interacts with cells within the bone marrow, thereby inducing tolerogenic training, modulating myelopoiesis, and driving the production of immunosuppressive MDSCs that are protective against lethal sepsis. Future studies aim to understand how these distinct responses are initiated, including investigations into the specific *C. dubliniensis*-PRR interactions, as well as the cytokine/receptor signaling axes that drive MDSC production and the TTI response in the bone marrow. Overall, the results presented here further our understanding of how the TTI response is initiated, which will ultimately allow for the development of novel immunotherapeutics and vaccine strategies to broadly target the inflammatory response during sepsis with timely suppression, as compared to traditional vaccine strategies that target specific pathogens.

## MATERIALS AND METHODS

### Strains, media, and growth conditions

The *C. dubliniensis* wild-type Wü284 strain was kindly provided by Gary Moran (68). The *C. albicans* DAY185 wild-type strain (a prototrophic derivative of SC5314) was a gift from Aaron Mitchel (69). The *C. tropicalis* wild-type strain (ATCC 44508) was obtained from the American Type Culture Collection (ATCC). The methicillin-resistant *S. aureus* strain (NRS383) was obtained from the Network on Antimicrobial Resistance in *S. aureus* data bank. Frozen stocks were maintained at −80°C and fungal and bacterial strains were freshly struck onto yeast peptone dextrose (YPD) or Trypticase soy agar, respectively, prior to use. Fungal strains were cultured in 10 mL of YPD broth with shaking (200 rpm) at 30°C for 12–18 h. *S. aureus* was initially cultured in 10 mL of Trypticase soy broth (TSB) with shaking (200 rpm) at 37°C overnight, then diluted 1:100 in fresh TSB and further cultured at 37C for 3 h until reaching log phase.

### Animals

Female Swiss Webster mice, 5–7 weeks of age, were purchased from Charles River Laboratories (NCI Cr:SW, strain 551). Female C57BL/6 mice (strain #000664), B6.129-*Card9^tm1Xlin^*/J mice (Card9 KO, strain #028652), and B6.129P2(SJL)-*Myd88^tm1.1Defr^*/J mice (Myd88 null, strain #009088), 5–7 weeks of age, were purchased from Jackson Laboratories. *Card9*-deficient mice were subsequently bred and maintained in-house. Previous studies evaluating sex as a biological variable with *C. dubliniensis* live immunization and polymicrobial challenge (as described below) showed similar levels of protection in male and female mice (E. A. Lilly and M. C. Noverr, unpublished data); therefore, female mice were used exclusively in these studies. Animals were housed and handled according to institutionally recommended guidelines. All experiments involving animals were approved by the Tulane University Institutional Animal Care and Use Committee.

### Animal experiments

The animal model of *C. dubliniensis* immunization (1.75 × 10^7^ cells/mouse), followed 14 days later by polymicrobial intra-abdominal infection (IAI) challenge with *C. albicans* (1.4–2.1 × 10^7^ cells/mouse) and *S. aureus* (8 × 10^7^ cells/mouse) was carried out as previously described by our laboratory (4, 6, 19, 20). Live immunization and IAI challenge were administered by IP inoculation. All mice were observed for morbidity (hunched posture, inactivity, ruffled fur, hypothermia) and mortality and scored using the modified Mouse Clinical Assessment Score for Sepsis (70) for 10 days following lethal sepsis challenge.

To deplete HSPCs following IP immunization, WT Swiss Webster mice were injected IV with 500 µg anti-mouse c-Kit antibody (clone B28; Bio X Cell BE0280) or rat IgG2b isotype control antibody (clone LTF-2; Bio X Cell BE0090) in 200 µL sterile pH 7.0 dilution buffer (Bio X Cell IP0070). Antibodies were given at 7 and 12 days post-immunization (7 and 2 days prior to lethal sepsis challenge). In subsequent experiments, WT C57BL/6 mice were injected IP with 500 µg anti-mouse c-Kit antibody (clone ACK2; Bio X Cell BE0293) or rat IgG2b isotype control antibody at the indicated time points post-immunization.

### Flow cytometry

To study the effect of *Candida* spp. on the bone marrow compartment, live *Candida* spp. (1.75 × 10^7^ cells/mouse, unless otherwise noted) were administered to mice by IP inoculation, as described above. Mice were euthanized by CO_2_ at the indicated time points following inoculation, and bone marrow cells were isolated and frozen for future use (71, 72). Peritoneal cell isolation was carried out as described previously (6).

For flow cytometry analysis, single cell suspensions (1–5 × 10^6^ cells) were stained with eFluor 506 Fixable Viability dye at a concentration of 1:1,000 in PBS for 30 min. The cells were then washed with FACS buffer (PBS, 2% heat-inactivated fetal bovine serum, 5 mM EDTA) and incubated with Fc block at a concentration of 1:100 in FACS buffer for 10 min on ice. Staining for HSPCs was carried out as described previously (13). Briefly, a cocktail of lineage antibodies (anti-TER119, anti-CD11b, anti-CD3e, anti-CD45R, anti-Ly-6G/C) was added for 30 min, followed by washing with FACS buffer, and a second incubation with anti-c-Kit, anti-Sca-1, anti-CD150, anti-CD48, anti-Flt3, and anti-CD34 for 30 min. Staining for committed progenitor (CPs) was adapted from reference 22; cells were blocked with anti-CD16/32-eF450 for 30 min, washed and incubated with the lineage cocktail as above, washed again, and finally incubated with anti-c-Kit, anti-Sca-1, anti-CD34, anti-Flt3, and anti-CD127 for 30 min. Staining for MDSCs was as described previously (6); cells were incubated with Fc block as above, followed by staining with either anti-CD11b and anti-Gr-1 (total MDSCs) or anti-CD11b, anti-Ly-6G, and anti-Ly-6C (M/G-MDSCs) for 30 min. All staining was carried out at 4°C and protected from light.

Following staining, cells were washed 2× with FACS buffer and fixed with 4% paraformaldehyde for 15 min on ice. Following fixation, cells were washed and resuspended in FACS buffer until analysis. In some experiments, CountBright Absolute Counting Beads (Invitrogen) were added to samples just before analysis. Cell analysis was carried out on a BD LSRFortessa Flow Cytometer (BD Biosciences), and the data were analyzed using FlowJo (Tree Star) Software. Unstained cells and UltraComp eBeads Compensation Beads (Invitrogen) stained with individual fluorophores were used to calculate compensation. Fluorescence minus one controls were included as gating controls. Antibody source and dilution information are detailed in Table 1 and representative gating strategies are illustrated in Fig. S2 and S4.

**TABLE 1:** Flow cytometry antibodies used in this study^*a*^

Purpose	Marker	Fluorophore	Clone	Dilution	Source	Cat #
Viability	Live/dead	eFluor506		1:1,000	eBioscience	65-0866-14
Block	CD16/32		93	1:100	eBioscience	14-0161-85
Block	CD16/32		2.4G2	1:100	BD	553142
Lin	CD3e	APC-Cy7	145-2C11	1:100	BD Pharmingen	561042
Lin	CD11b	APC-Cy7	M1/70	1:100	BD Pharmingen	561039
Lin	CD45R	APC-Cy7	RA3-6B2	1:100	BD Pharmingen	552094
Lin	Ly-6G/Ly-6G (Gr-1)	APC-Cy7	RB6-8C5	1:100	BD Pharmingen	557661
Lin	TER-119	APC-Cy7	TER-119	1:100	BD Pharmingen	560509
LKS	c-Kit (CD117)	APC	2B8	1:100	eBioscience	17-1171-81
LKS	Sca-1 (Ly6A/E)	PE-Cy7	D7	1:100	eBioscience	25-5981-81
HSPC	CD150	eFluor450	mShad150	1:100	eBioscience	48-1502-82
HSPC	CD48	PerCP-eFluor710	HM48-1	1:100	eBioscience	46-0481-80
HSPC/CP	Flt3 (CD135/Flk2)	PE	A2F10.1	1:100	BD Pharmingen	561068
HSPC/CP	CD34	FITC	RAM34	1:100	eBioscience	11-0341-81
CP/Block	CD16/32	eF450	93	1:100	eBioscience	48-0161-80
CP	IL-7Rα (CD127)	PerCP-eFluor710	A7R34	1:100	eBioscience	46-1271-80
MDSC	CD11b	Alexa Fluor 488	M1/70	1:100	BD	557672
MDSC	Ly-6G/Ly-6C (Gr-1)	PE	RB6-8C5	1:100	BD	553128
MDSC	Ly-6G	PE-Cy7	1A8	1:100	BD	560601
MDSC	Ly-6C	PE	AL-21	1:100	BD	560592

^*a*^ Lin, lineage; LKS, Lineage^−^ c-Kit^+^ Sca-1^+^; HSPC, hematopoietic stem and progenitor cells; CP, committed progenitors; MDSC, myeloid-derived suppressor cells.

### Immunoassays

Bone marrow extracellular fluid was collected from naïve and immunized mice (2 dpi) as previously described (22). Briefly, femoral bones were flushed with 500 uL cold PBS, the cells were pelleted by centrifugation at 500 × *g* for 5 min, and the supernatant was collected and frozen at −80°C until analysis. Bone marrow supernatants were analyzed using the Luminex-based Milliplex MAP Mouse Cytokine/Chemokine Magnetic Bead Panel (MCYTMAG-70K-PX32) according to the manufacturer’s instructions. Data were analyzed using Bio-Plex Manager software (Bio-Rad).

Peritoneal flushes were carried out as previously described (73), and peritoneal lavage fluid (PLF) was collected and cleared by centrifugation at 10,000 rpm for 10 min, followed by freezing at −80°C until analysis. IL-6 was measured using a mouse IL-6 ELISA MAX kit (Biolegend), according to the manufacturer’s instructions.

### Bone marrow infiltration

Fungal burden in the bone marrow was enumerated by spot plating serial dilutions of bone marrow suspensions onto YPD agar as described previously (7).

### Statistics

Comparisons between two groups were assessed by a Student’s *t*-test. Comparisons between multiple groups were assessed by an analysis of variance (ANOVA) with Dunnett’s (when comparing to a control mean) or Tukey’s (when comparing multiple sets of means) multiple comparisons test. Survival curves were compared using the log-rank test with Holm-Sidak multiple comparisons test. Significant differences were defined at a *P* value of <0.05. All statistical analyses were performed using GraphPad Prism software.

## SUPPLEMENTAL MATERIAL

Fig. S1 mbio.02906-25-s0001.tif Survival following c-Kit depletion and lethal sepsis challenge.

Fig. S2 mbio.02906-25-s0002.tif Gating strategy for HSPCs in the bone marrow.

Fig. S3 mbio.02906-25-s0003.tif Additional bone marrow HSPC phenotyping by flow cytometry.

Fig. S4 mbio.02906-25-s0004.tif Gating strategy for putative MDSCs in the bone marrow.

Fig. S5 mbio.02906-25-s0005.tif Additional bone marrow cytokines.

Supplemental Figure Legends mbio.02906-25-s0006.docx Legends for Fig. S1-S5.

ASM does not own the copyrights to Supplemental Material that may be linked to, or accessed through, an article. The authors have granted ASM a non-exclusive, world-wide license to publish the Supplemental Material files. Please contact the corresponding author directly for reuse.
