# Supplementary figures and images for: Induction of myelopoiesis by Candida dubliniensis drives protective trained immunity against sepsis in a Card9-dependent manner
Source: mBio. 2025 Oct 31;16(12):e02906-25. doi: 10.1128/mbio.02906-25 (PMC12691594; doi:10.1128/mbio.02906-25)

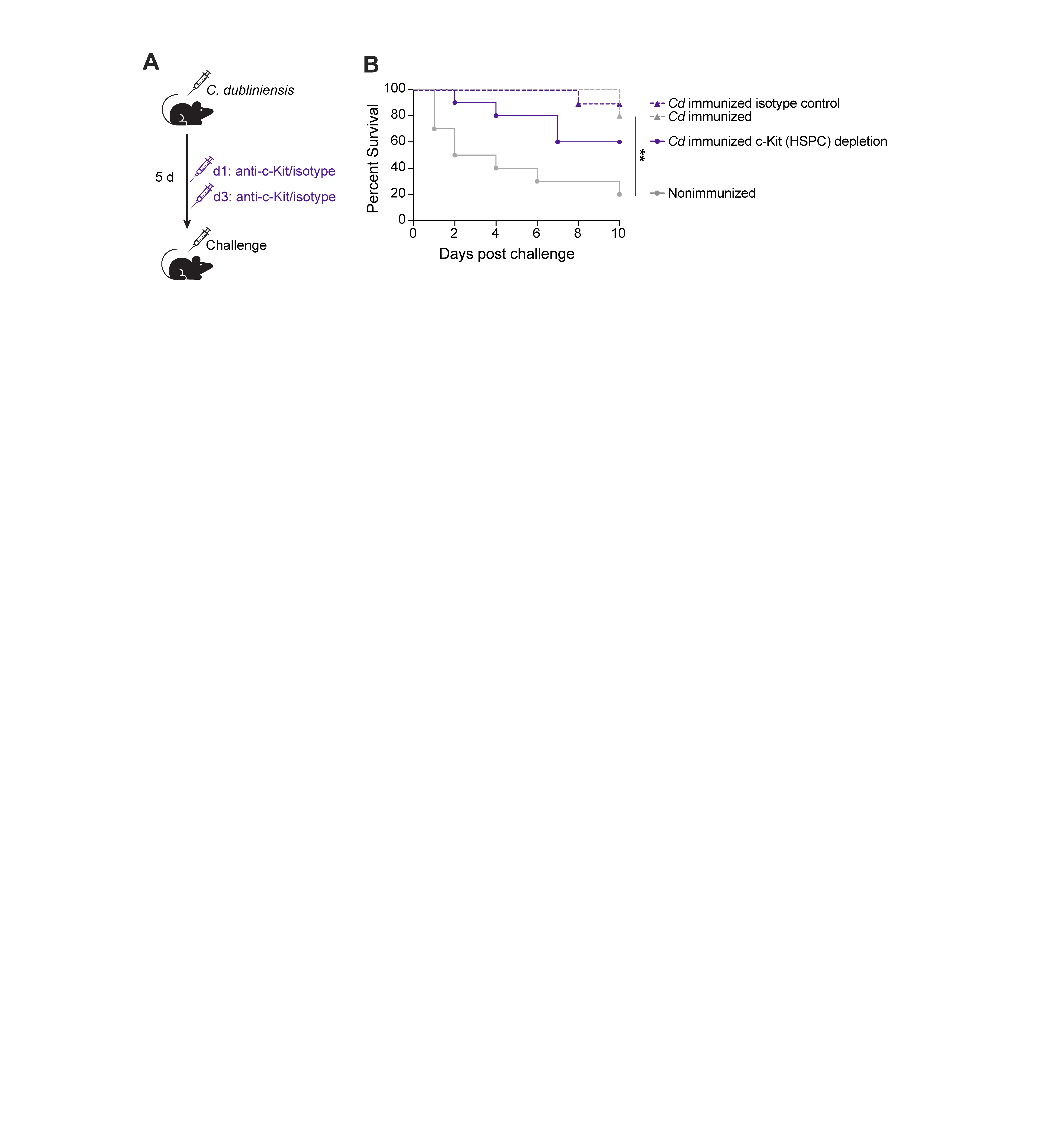

Supplement: Fig. S1 — Survival following c-Kit depletion and lethal sepsis challenge. [file mbio.02906-25-s0001.tif]

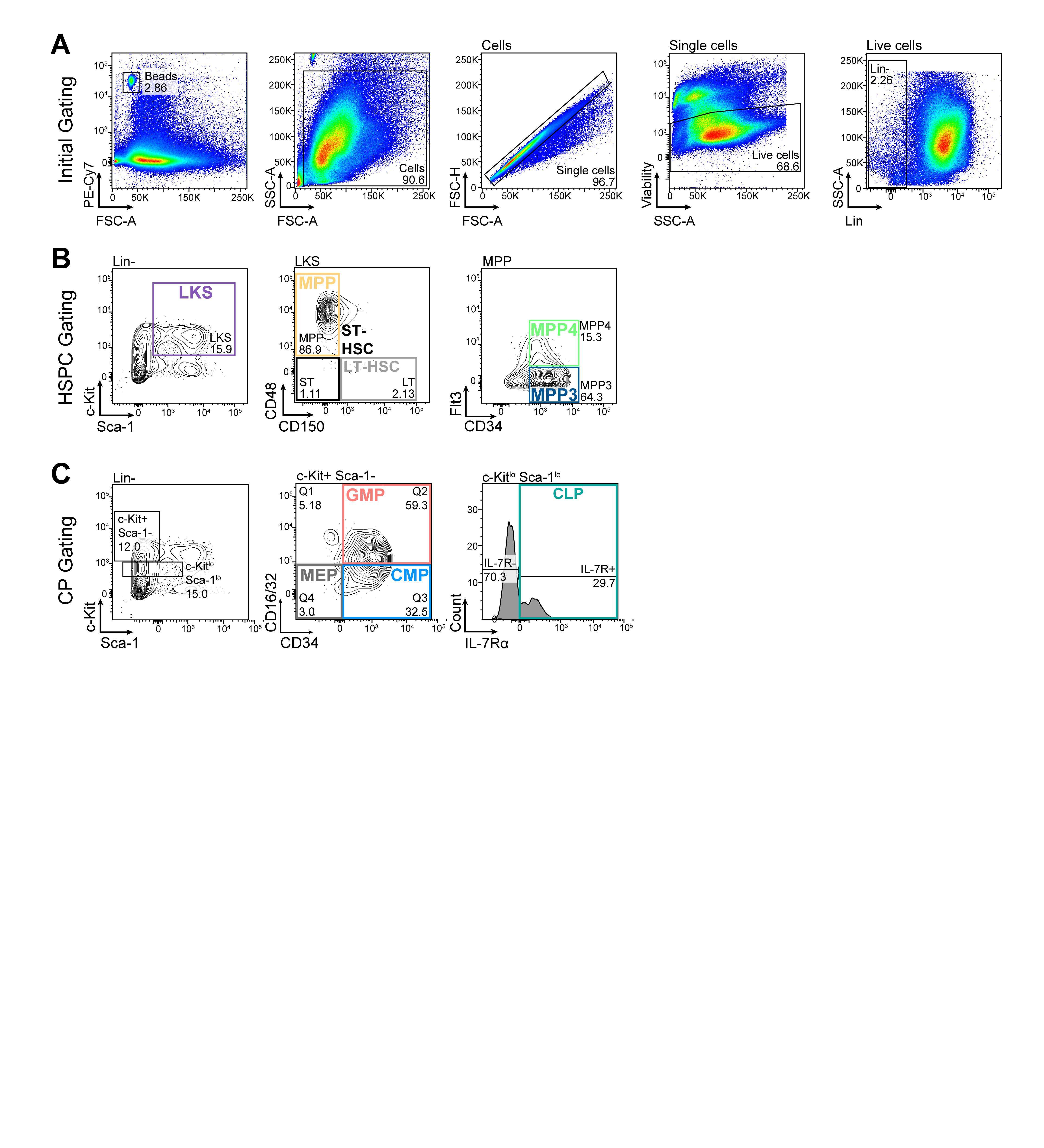

Supplement: Fig. S2 — Gating strategy for HSPCs in the bone marrow. [file mbio.02906-25-s0002.tif]

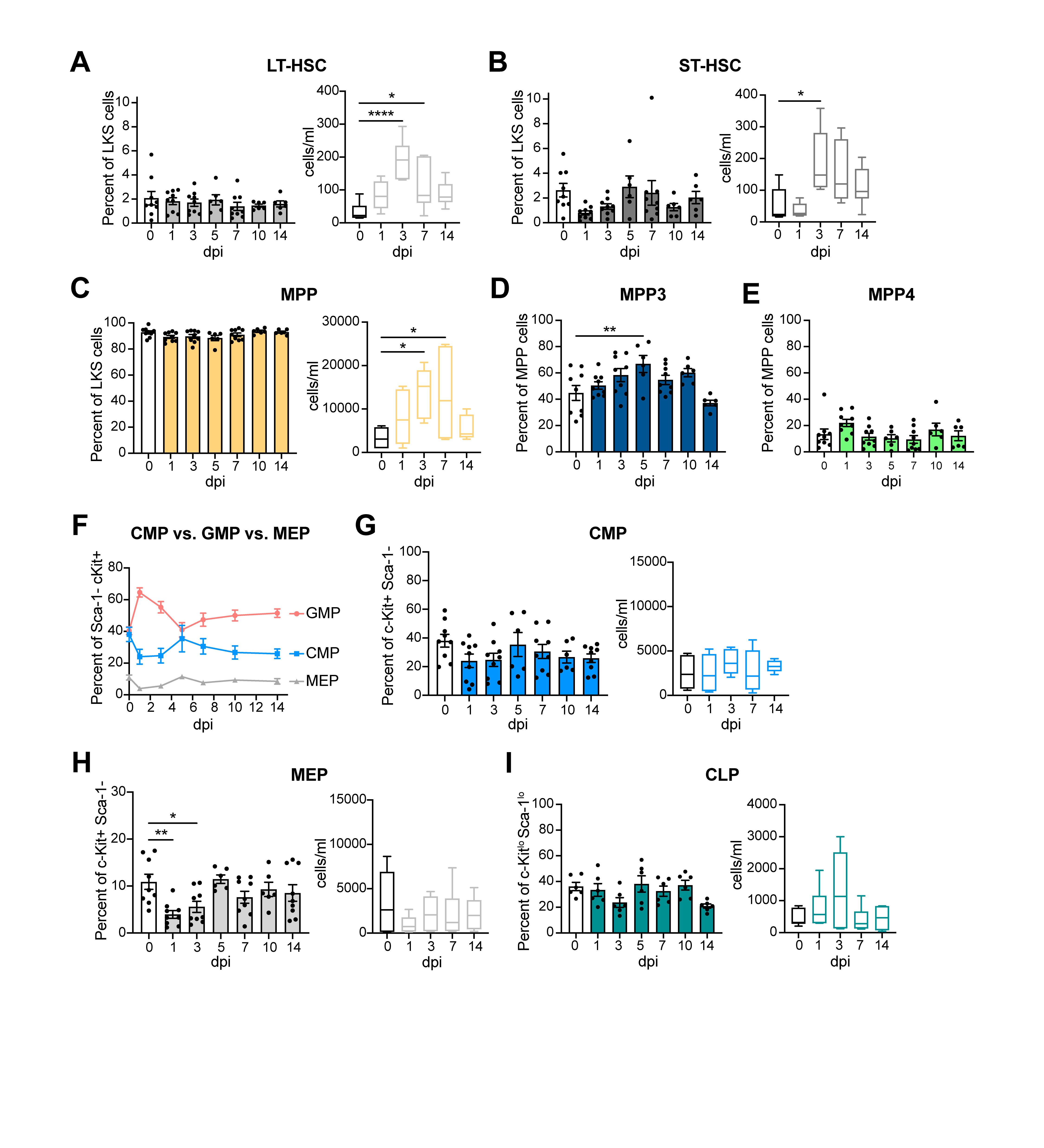

Supplement: Fig. S3 — Additional bone marrow HSPC phenotyping by flow cytometry. [file mbio.02906-25-s0003.tif]

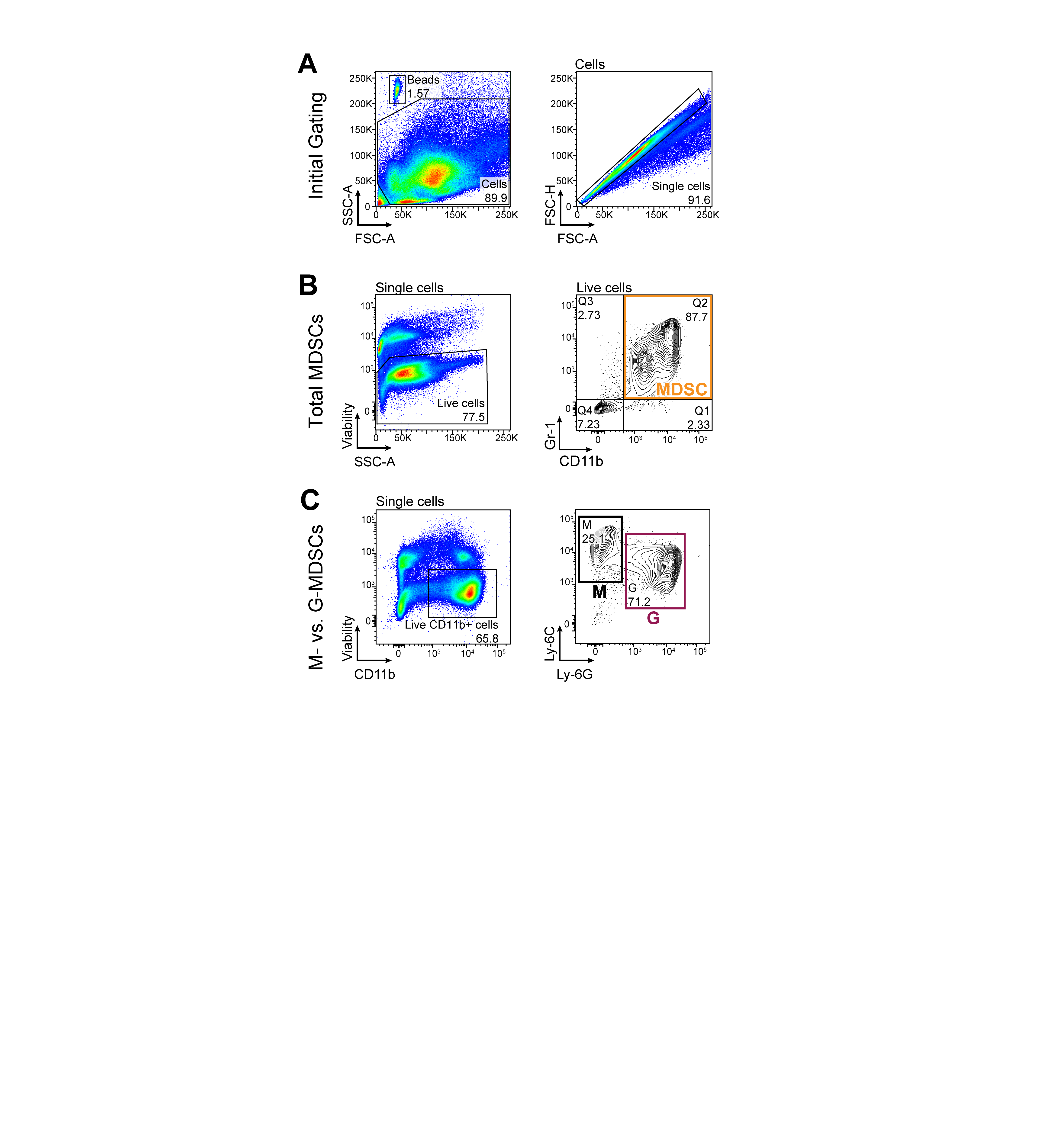

Supplement: Fig. S4 — Gating strategy for putative MDSCs in the bone marrow. [file mbio.02906-25-s0004.tif]

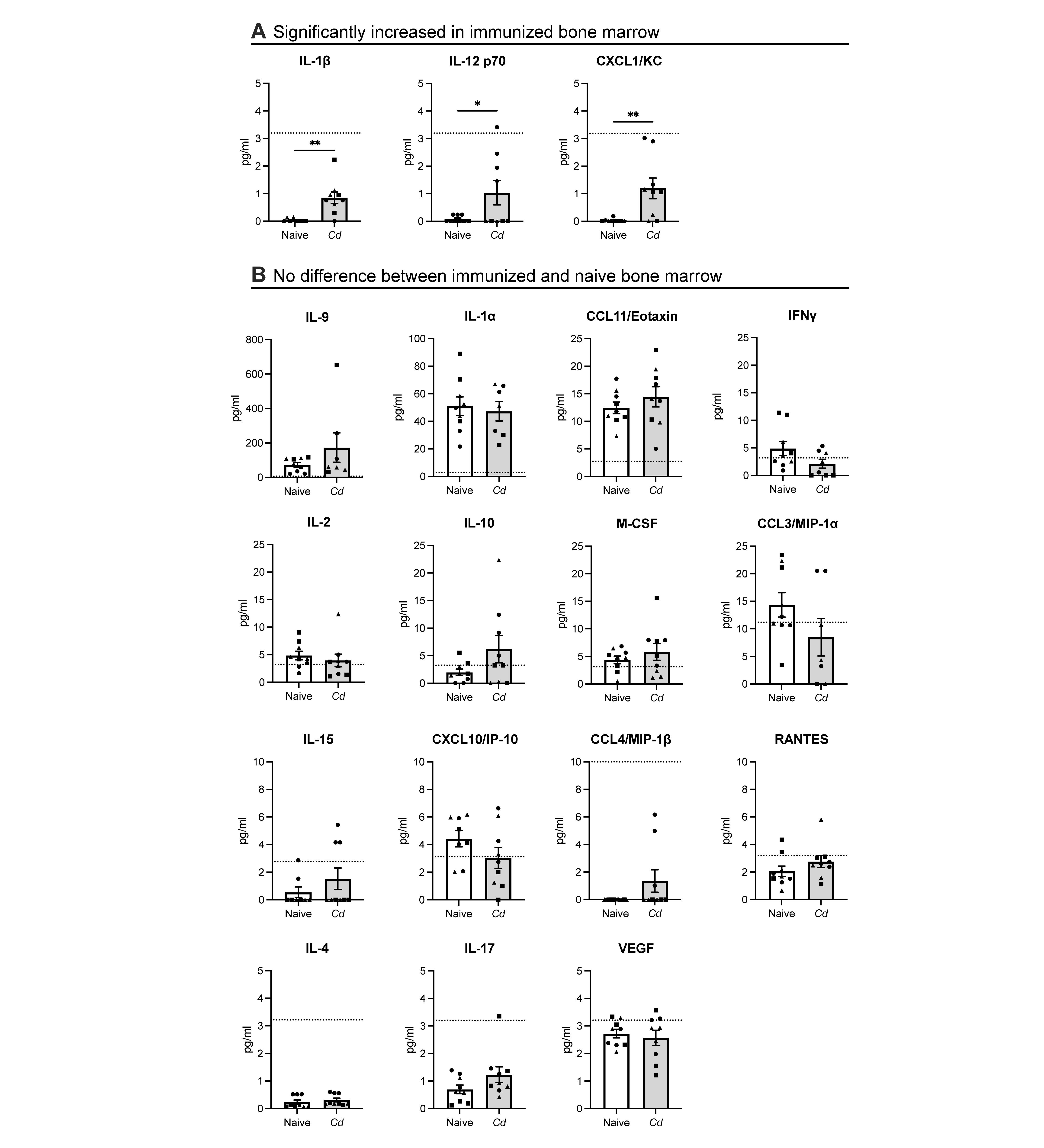

Supplement: Fig. S5 — Additional bone marrow cytokines. [file mbio.02906-25-s0005.tif]
