# Supplementary material for: Induction of myelopoiesis by Candida dubliniensis drives protective trained immunity against sepsis in a Card9-dependent manner
Source: mBio. 2025 Oct 31;16(12):e02906-25. doi: 10.1128/mbio.02906-25 (PMC12691594; doi:10.1128/mbio.02906-25)
Supplement: Supplemental Figure Legends — Legends for Fig. S1-S5. [file mbio.02906-25-s0006.docx]

**SUPPLEMENTAL FIGURE LEGENDS**

**Figure S1.** (**A**) Experimental design: WT C57BL/6 mice were immunized IP with 1.75x10^7^ *C. dubliniensis* cells, followed by IP administration of 500 µg of anti-c-Kit (clone ACK2) or isotype control antibody on d1 and d3, and lethal IP sepsis challenge on d5. (**G**) Survival following c-Kit (HSPC) depletion and lethal sepsis challenge. *n*=10/group. ***p*<0.01, log-rank test with Holm-Sidak multiple comparisons test.

**Figure S2.** Gating strategy for hematopoietic stem and progenitor cells (HSPCs) in the bone marrow, related to Fig. 1B-C and Fig. 2. (**A**) **Initial gating:** Counting beads were gated in FSC-A vs. PE-Cy7; cells were gated in FSC-A vs. SSC-A; singlets were gated using FSC-A vs. FCS-H; live cells were gated in SSC-A vs. viability stain; lineage negative (Lin^-^) cells were gated in lineage markers vs. SSC-A. (**B**) **Hematopoietic stem and progenitor cell (HSPC) gating:** the lineage negative population was gated on c-Kit and Sca-1 and LKS cells were defined as double positive for c-Kit and Sca-1; the LKS population was further gated on CD150 and CD48, and long-term (LT)-HSCs were defined as CD150^+^ CD48^-^, short-term (ST)-HSCs were defined as CD150^-^ CD48^-^, and multipotent progenitor cells (MPPs) were defined as CD150^-^ CD48^+^; the MPP population was further gated on CD34 and Flt3, and MPP3 and MPP4 populations were defined as CD34^+^ Flt3^-^ and CD34^+^ Flt3^+^, respectively. (**C**) **Committed progenitor (CP) gating:** the lineage negative population was gated on c-Kit and Sca-1; the c-Kit^+^ Sca-1^-^ population was further gated on CD34 and CD16/32, and granulocyte-macrophage progenitor (GMP) cells were defined as CD34^+^ CD16/32^+^, common myeloid progenitor (CMP) cells were defined as CD34^+^ CD16/32^-^, and megakaryocyte-erythrocyte progenitor (MEP) cells were defined as CD34^-^ CD16/32^-^; the c-Kit^lo^ Sca-1^lo^ population was further gated on IL-7Rα, and common lymphoid progenitor (CLP) cells were defined as IL-7Rα^+^.

**Figure S3.** Additional bone marrow HSPC phenotyping by flow cytometry. Bone marrow cells were isolated as described in Fig. 2. (**A**) LT-HSCs (LKS CD150^+^ CD48^-^). (**B**) ST-HSCs (LKS CD150^-^ CD48^-^). (**C**) MPPs (LKS CD150^-^ CD48^+^). (**D**) MPP3 (LKS CD150^-^ CD48^+^ CD34^+^ Flt3^-^). (**E**) MPP4 (LKS CD150^-^ CD48^+^ CD34^+^ Flt3^+^). (**F**) CMP (Lin^-^ c-Kit^+^ Sca-1^-^ CD34^+^ CD16/32^-^) vs. GMP (Lin^-^ c-Kit^+^ Sca-1^-^ CD34^+^ CD16/32^-^) vs. MEP (Lin^-^ c-Kit^+^ Sca-1^-^ CD34^-^ CD16/32^-^). (**G**) CMPs. (**H**) MEPs. (**I**) CLPs (Lin^-^ c-Kit^lo^ Sca-1^lo^ IL-7Rα^+^). Cell percentages of parent population (left; *n*=6-9/group, combined from 2-3 experiments) and cell numbers expressed as cells/ml (right; *n*=6/group, combined from 2 experiments). **p*<0.05, ***p*<0.01, *****p*<0.0001; one-way ANOVA with Dunnett’s multiple comparisons test. Data are mean ± s.e.m (middle) or boxplots showing 25-75 percentiles, medians and whiskers spanning min-max (right). Representative gating strategies illustrated in Fig. S2.

**Figure S4.** Gating strategy for putative MDSCs in the bone marrow, related to Fig. 3. (**A**) **Initial gating:** Counting beads and cells were gated in FSC-A vs. SSC-A; singlets were gated using FSC-A vs. FSC-H. (**B**) **Total putative MDSCs**: Live cells were gated in SSC-A vs. viability stain; live cells were further gated on CD11b and Gr-1, and the total putative MDSC population was defined as double positive for CD11b and Gr-1. (**C**) **M- vs. G-MDSCs**: Single cells were gated on CD11b and viability staining, and live CD11b^+^ cells were further gated on Ly-6G and Ly-6C; M-MDSCs were defined as Ly-6G^-^ Ly-6C^hi^ and G-MDSCs were defined as Ly-6G^+^ Ly-6C^+^.

**Figure S5.** Additional bone marrow cytokines. Bone marrow supernatants were collected and analyzed as described in Fig. 4. Additional cytokines that were (**A**) significantly increased in immunized bone marrow or (**B**) displayed no difference between immunized and naïve bone marrow. *n*=9/group, combined from 3 experiments (symbols represent independent experiments). **p<*0.05, ***p<*0.01, two-tailed t-test. Data are mean ± s.e.m. Dashed line indicates lower limit of detection. Additional cytokines measured but not included due to all values being at or below the lower limit of detection include the following: GM-CSF, IL-3, IL-5, IL-6, IL-7, IL-12 p40, LIF, LIX, TNFα.
